# The Position of DNA Cleavage by TALENs and Cell Synchronization Influences the Frequency of Gene Editing Directed by Single-Stranded Oligonucleotides

**DOI:** 10.1371/journal.pone.0096483

**Published:** 2014-05-01

**Authors:** Natalia Rivera-Torres, Bryan Strouse, Pawel Bialk, Rohina A. Niamat, Eric B. Kmiec

**Affiliations:** Delaware State University, Department of Chemistry, Dover, Delaware, United States of America; Saint Louis University, United States of America

## Abstract

With recent technological advances that enable DNA cleavage at specific sites in the human genome, it may now be possible to reverse inborn errors, thereby correcting a mutation, at levels that could have an impact in a clinical setting. We have been developing gene editing, using single-stranded DNA oligonucleotides (ssODNs), as a tool to direct site specific single base changes. Successful application of this technique has been demonstrated in many systems ranging from bacteria to human (ES and somatic) cells. While the frequency of gene editing can vary widely, it is often at a level that does not enable clinical application. As such, a number of stimulatory factors such as double-stranded breaks are known to elevate the frequency significantly. The majority of these results have been discovered using a validated HCT116 mammalian cell model system where credible genetic and biochemical readouts are available. Here, we couple TAL-Effector Nucleases (TALENs) that execute specific ds DNA breaks with ssODNs, designed specifically to repair a missense mutation, in an integrated single copy eGFP gene. We find that proximal cleavage, relative to the mutant base, is key for enabling high frequencies of editing. A directionality of correction is also observed with TALEN activity upstream from the target base being more effective in promoting gene editing than activity downstream. We also find that cells progressing through S phase are more amenable to combinatorial gene editing activity. Thus, we identify novel aspects of gene editing that will help in the design of more effective protocols for genome modification and gene therapy in natural genes.

## Introduction

Single base mutations can be repaired by the introduction of DNA oligonucleotides (ssODN) into a target cell [1]–[3]. The frequency of this corrective activity depends on a number of factors including the length of ssODN, the position of the cell in its proliferative cycle [4]–[5] and the presence of double-stranded DNA breaks in the host genome [6]–[7]. Studies centered on the effect of S phase transition on gene repair have led to the emergence of a model in which reversal of genotype takes place most often through the incorporation of the ssODN into a newly synthesized DNA strand [1]. The level of gene repair is enhanced dramatically when cells are targeted during S phase and, specifically, when they are slowed in their progression through S phase [8]–[10].

Anticancer drugs were tested as agents to stimulate gene editing activity based on the concept that DNA damage (in the form of ds DNA breaks) might activate proteins involved in homology directed repair, slow cell cycle progression and thus stimulate gene correction. Ferara *et al* [11] demonstrated that pre-treatment of cells targeted for gene editing by ssODNs with camptothecin (CPT) enhanced gene editing activity 5–10 fold. But, such treatment of cells leads to nondiscriminate, nonspecific ds breaks which again presents a practical barrier in the development of gene editing for molecular medicine.

One solution to this problem appears to lie in the use of enzymes that can produce a “unique” specific ds DNA break in the genome, preferably at or near the position of the mutant base. Although the field is evolving, three major agents are currently used to catalyze specific ds DNA breaks: Zinc-Finger Nucleases (ZFNs), Clustered Regularly Interspersed Short Palindromic Repeats (CRISPR-Cas9) and Transcription Activator-Like Effector Nucleases (TALENs) [12]–[17]. By using agents that cut specifically, one can reduce the chance of offsite mutations while simultaneously stimulating the frequency of gene editing. These so-called “programmable nucleases” [18] may enable the more efficient use of the ds break as a stimulatory factor in reactions designed to correct single base mutations.

We have chosen to utilize TALENs to enhance the frequency of gene editing, directed by ssODNs, and repair a missense mutation in the eGFP gene [19]. A single copy is integrated into the genome of a clonally isolated and expanded HCT116–19 cell line [19]–[20]. This well-established model system has unique advantages including the capacity to correlate genotypic and phenotypic changes with functional protein activity. Recently, we showed that the combinatorial action of ssODNs and a TALEN designed to cut at −2/−3 relative to the mutant base (**G→C**) results in a substantial rise in the frequency of gene editing [21]. Importantly, TALENs reduce the level of ssODNs needed for nucleotide exchange, eliminating the onset of the Reduced Proliferation Phenotype (RPP) [22]. TALENs and ssODNs had been reported previously to work together to facilitate genome editing [18] [23]–[28], but we have taken a more decidedly reductionist approach to characterize this reaction in somatic cells. In this paper, we build upon that original observation and focus on *(1)* the position of the cut site in the region surrounding the mutant base, and *(2)*, the effect of cell synchronization [29]–[31] on specific TALEN activity to enable gene editing in somatic cells.

## Materials and Methods

### Cell Line and Culture Conditions

HCT116 cells were acquired from ATCC (American Type Cell Culture, Manassas, VA). HCT116–19 cell line was created by integrating a pEGFP-N3 vector (Clontech, Palo Alto, CA) containing a mutated eGFP gene. The mutated eGFP gene has a nonsense mutation at position +67 resulting in a nonfunctional eGFP protein. For these experiments, HCT116 (−19) cells were cultured in McCoy’s 5A Modified medium (Thermo Scientific, Pittsburgh, PA) supplemented with 10% fetal bovine serum, 2 mM L-Glutamine, and 1% Penicillin/Streptomycin. Cells were maintained at 37°C and 5% CO_2_. Custom designed oligonucleotides, 72NT, 40NT and 100NT were synthesized from IDT (Integrated DNA Technologies, Coralville, IA).

### TALEN Design and Construction

Nine Left and eleven Right TALEN half-sites were designed to flank the target at a range of −39 to +46 base pairs of the integrated mutant eGFP gene (TA**G** = 0). TALENs were designed according to previously published guidelines [32] to have a Thymine (T) at position 0 of the TALEN binding sequence and a DNA binding domain of 15–20 base pairs (15–20 RVDs). The 20 constructed TALEN half-sites were combined for targeting experiments if the two half-sites produced a spacer between 13 and 29 base pairs. By using all possible combinations of TALEN half-sites, 22 total TALEN combinations were tested that flank the mutant base. Construction was done via the Gold Gate Assembly method originally developed by Cermak et al. [32] and purchased through Addgene (Addgene, Cambridge, MA). The final step of the assembly protocol was modified to include the mammalian expression vector pc-GoldyTALEN, which has been optimized for expression and cutting efficiency in mammalian systems [27]. Following construction, colony PCR and DNA sequencing by Genewiz Incorporated (South Plainfield, NJ) was performed to confirm correct TALEN constructs. The only differences between each TALEN half-site are the order in which the RVDs were arranged, corresponding to the DNA they were designed to target. The RVDs used were HD, NI, NG, and NN only. Following construction, colony PCR and DNA sequencing by Genewiz Incorporated (South Plainfield, NJ) was performed to confirm correct TALEN constructs (for reference, the full sequence of L848–19 TALEN plasmid can be seen in File S2).

### Transfection of HCT116–19 Cells and Experimental Approach

For experiments utilizing synchronized cells, HCT116–19 cells were seeded at 2.5×10^6^ cells in a 100 mm dish and synchronized with 6 µM aphidicolin for 24 hours. Cells were released for 4 hours prior to trypsinization and transfection by washing with PBS (−/−) and adding complete growth media. Synchronized and unsynchronized HCT116–19 cells were transfected at a concentration of 5×10^5^ cells/100 ul in 4 mm gap cuvette (BioExpress, Kaysville, UT). Single-stranded oligonucleotides and TALEN plasmid constructs were electroporated (250 V, LV, 13 ms pulse length, 2 pulses, 1 s interval) using a Bio-Rad Gene Pulser XCellTM Electroporation System (Bio-Rad Laboratories, Hercules, CA). Cells were then recovered in 6-well plates with complete growth media at 37°C for 48 hours prior to analysis, unless otherwise noted.

### Analysis of Gene Edited Cells

Fluorescence (eGFP) was measured by a Guava EasyCyte 5HT Flow Cytometer (Millipore, Temecula, CA). Cells were harvested by trypsinization, washed once with 1x PBS (−/−) and resuspended in buffer (0.5% BSA, 2 mM EDTA, 2 µg/mL Propidium Iodide (PI) in PBS −/−). Propidium iodide was used to measure cell viability as such, viable cells stain negative for PI (uptake). Correction efficiency was calculated as the percentage of the total live eGFP positive cells over the total live cells in each sample. Error bars are produced from two sets of data points generated over two separate experiments using basic calculations of Standard Error.

### TALEN Cleavage Analysis

HCT116–19 cells were electroporated at a concentration of 5×10^5^ cells/100 ul in 4 mm gap cuvette (BioExpress, Kaysville, UT) with TALEN pairs −35, −28, −1/+1 and +7/8 at 2 ug and 10 ug. Cells were then recovered in 6-well plates with complete growth media at 37°C for 72 hours. DNA was isolated using the Blood and Tissue DNeasy kit (Qiagen, Hilden, Germany). RFLP analysis was performed on 181 bp amplicons that were created using forward primer, 5′GAGGGCGATGCCACCTACGGC and reverse primer, 5′GGACGTAGCCTTCGGGCATGGC. PCR samples were cleaned up using the QIAquick PCR purification kit (Qiagen, Hilden, Germany) and treated with the indicated restriction enzymes following the manufactures protocol. Digested samples were loaded along with NEB 2-log DNA ladder (NEB, Ipswich, MA) into a 2% TBE agarose gel for analysis. T7 Endonuclease assay was performed on amplicons of 605 bp with forward primer 5′CTGGACGGCGACGTAAACGGC and reverse primer, 5′ACCATGTGATCGCGCTTCTCG. Following PCR cleanup, each TALEN treated sample was placed in a thermocycler for heteroduplex formation. Samples were then treated with 1 ul of T7 endonuclease at 37°C for 1 hour and then subjected to electrophoresis on a 2% TBE agarose gel containing ethidium bromide. Images for both RFLP and T7 assay were collected by the Gel Doc EZ System (BioRad, Hercules, CA).

## Results and Discussion

### Results

**Figure 1:**
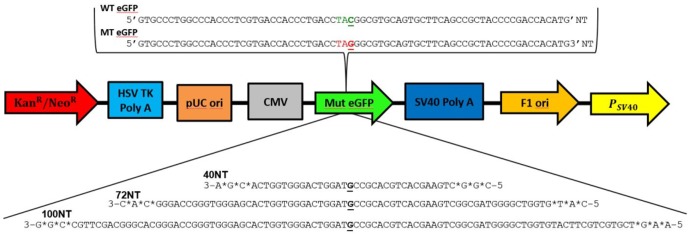
Gene editing model system and ssODNs. The wild-type and mutated eGFP gene segments with the target codon located in the center of the sequences are displayed in green and red respectively. The nucleotide targeted for exchange is emphasized in bold and underlined. Phosphorothioate modified, end protected (denoted with *) 40NT, 72NT, 100NT a 40-mer 72-mer and 100-mer which are used to target the non-transcribed (NT) strand are shown below. Also depicted is the sequence of the 72T, which directs exchange on the transcribed strand (T) of the mutated eGFP gene.

The eGFP gene was mutated near the 5′ end of the coding sequence creating a stop codon (TAG) in place of a tyrosine (TAC). As such, eGFP is produced in truncated form and no green fluorescence is observed in the cell. Figure 1 displays some of the target sequence and the integration vector used to insert a single copy of the gene driven by a CMV promoter in HCT116 cells [19]. Also displayed are three ssODNs that are designed to create a mismatch with the G residue of the TAG codon on the nontranscribed strand (NT) (*or sense or nontemplate strand*), thereby directing gene editing at this base. The three NT-ssODNs are of identical sequence through 40 bases but vary in length from 40 to 72 to 100 nucleotides respectively. This system is well established as a model for analyzing the mechanism of gene editing in human cells [9] [29]–[30]. In most applications, 72NT ssODN has been used in optimization studies for delivery and the response of cell and genomic DNA in the gene editing reaction [20]. Once repair of the TA**G→**TA**C** has been facilitated, the population of cells is analyzed by FACS and the percentage of live green fluorescent cells within that population can be presented as the frequency of gene correction. Sorted eGFP^+^ cells are easily quantified and genotype verified by direct DNA sequencing. Thus, genotype and phenotype, expression of a functional protein, is assessed in a valid, simple way, a critical component of reaction optimization or characterization studies.

**Figure 2:**
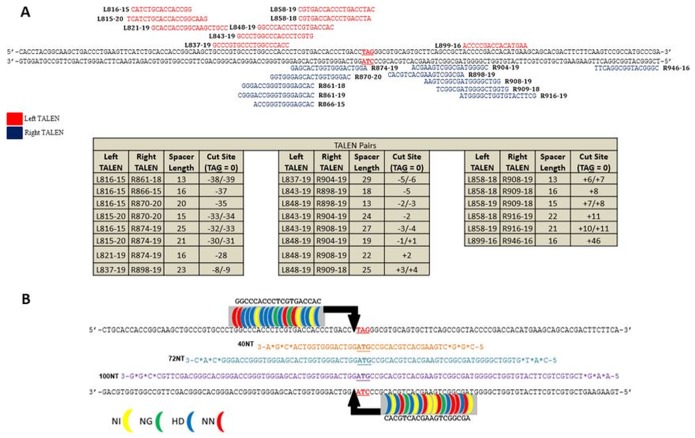
Array of TALENs Designed for Proximal Cleavage Analysis. (**A**) Nine Left and eleven Right TALEN half-sites were designed to flank the target at a range of −39 to +46 base pairs of the integrated mutant eGFP gene (TA**G** = 0). TALENs were designed according to previously published guidelines (see materials and methods). The 20 constructed TALEN half-sites, 9 left depicted in red, and 11 right depicted in blue, were combined for targeting experiments. By using all possible combinations of TALEN half-sites with spacers between 13–29 bases; 22 total TALEN combinations were tested that flank the mutant base. Cut site reflects the predicted position of ds break induced by the TALEN pair relative to the mutant base (TA**G**). (**B**) The TALEN pair, designed and built using the Golden Gate method, induces a double stranded break immediately preceding the mutant codon. RVDs are shown as color coded binding blocks next to their respective base, yellow NI:A, green NG:T, blue HD:C and red NN:G. Fok1 domains are shown in black and are positioned at their predicted cut site.

We have shown previously that the gene editing directed by 72-mers (NT) take place at an approximate level of 0.7% unless synchronized and released cells are used; in that case, frequencies approach 2% [8] [31]. In either case, however, in past studies, the level of ssODN required to activate the reaction is so high that the corrected cells cease to proliferate; a phenomenon we termed, Reduced Proliferation Phenotype [22]. By incorporating TALENs into the reaction mixture, we were able to reduce the amount of ssODN in the reaction and corrected cells responded by continuing their normal growth rate [21]. The TALEN pair used in those studies cuts the eGFP gene 5′ (upstream) of the target base. Since TALENs can be “programmed” to cleave at most sites in the DNA, we created an array of TALENs to analyze the impact of the cut site on gene editing of the TA**G** codon. The 20 TALEN plasmids (9 Left and 11 Right: Figure 2A) were designed according to previously published guidelines and recommendations [32]. Briefly, each TALEN half-site binding sequence (left or right plasmid) is preceded by a thymine (T) and contains 15–20 RVDs which bind to the DNA sequence; 15–20 base pairs respectively. Through this strategy, 22 TALEN combinations were created. These combinations allowed for the creation of TALENs with a range of spacer sizes which can dictate or even restrict the position of cleavage sites relative to the mutant base. Cloning of the TALEN constructs was done via the Golden Gate Assembly method originally developed by Cermak *et al* [32] with the final step of the assembly protocol modified to include the mammalian expression vector pc-GoldyTALEN [27].

**Figure 3:**
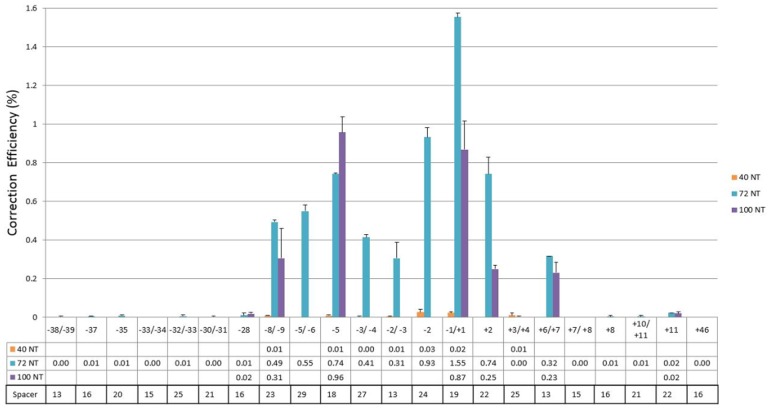
Fine Mapping of TALEN Cleavage site and Gene Editing Activity. Unsynchronized HCT116–19 cells were harvested and electroporated at a concentration of 5e5 cells/100 ul with TALENs and the corresponding ODN (40NT, 72NT or 100NT ODN) under the standard reaction ratio TALEN:ssODN (2 µg/1.35 µg). TALEN amounts reflect the total TALEN plasmid added to each sample in equal portions. Following electroporation, cells were placed in 6-well plates and allowed to recover for 48 hours. Analyses took place on a Guava EasyCyte 5HT flow cytometer (see Materials and Methods). Correction efficiency (%) was determined by the number of viable eGFP positive cells divided by the total viable cells in the population. Each treatment was performed in duplicate and error bars represent standard error.

Thus, the experimental protocol was to introduce ssODN and two plasmids (one expressing the left and one, the right TALEN) into HCT116–19 cells, a clonally expanded line that contains a single copy of the mutant eGFP gene and at various times thereafter, analyze gene editing activity by FACS. The goal is to define the range of genomic cleavage sites that enable gene editing directed by ssODNs bearing different lengths. We had already established that the reaction was dependent on both TALEN arms being present (Figure 2B), the presence of a specific ssODN designed to direct the change and an optimized TALEN: ssODN ratio [21]. Three different lengths of ssODN, all complimentary to the sense or non-template strand (NT) were used; 40NT, 72NT and 100NT. The cut sites are predicted to occur at the center of the spacer region for each TALEN pair. In our system, cut sites are designated by their position relative to the target base (G) in the TA**G** codon. For example, where **G** is 0, the 5′ end of **G** is −1, the 3′ end is +1, and so on. When the spacer region of the TALEN pair contains an even number of nucleotides, there is one predicted cut site. When the spacer region contains an odd number of nucleotides, there are two predicted cut sites because with even spacers, the cut site falls directly between two bases in the spacer region while with odd numbered spacer regions, the center falls directly on a unique nucleotide. Consequently, the predicted cleavage can occur on either side of the nucleotide at the center of the spacer region. For example, the TALEN pair L848–19 and R898–19 has a spacer length of 13 nucleotides. The direct center of this spacer region does not fall between two nucleotides but rather on *top* of the 7^th^ nucleotide. Accordingly, the cleavage is predicted to occur on either side of this nucleotide, which is either −2 or −3 from the TA**G**. Following electroporation, cells were placed in 6-well plates and allowed to recover for 48 hours and fluorescence detected by FACS. The results (Figure 3) reveal significant gene editing activity when TALEN activity takes place, −8/−9 bases upstream to +6/+7 bases downstream respectively, relative to the target base (0). In an attempt to expand the range of effective cleavage sites, we utilized a 100-mer (100NT), that would hybridize further upstream and downstream; TALEN pairs that cut at sites −28, −8/−9, −5, −1/+1, +2, +6/+7 and +11 were tested. None of the cut sites enabled higher activity when the 100-mer was used as compared to using 72NT. Past studies had shown low activity with shorter ssODNs, but to complete the analyses, 40NT was also tested with a selection of TALENs that cleave at −8/−9, −5, −3/−4, −2/−3, −2, −1/+1 and +3/+4 respectively. The 40NT displayed nearly undetectable gene editing activity throughout the broad range of target sites consistent with previous observations [33].

### Cell Synchronization in TALEN/ssODN-directed Gene Editing

**Figure 4:**
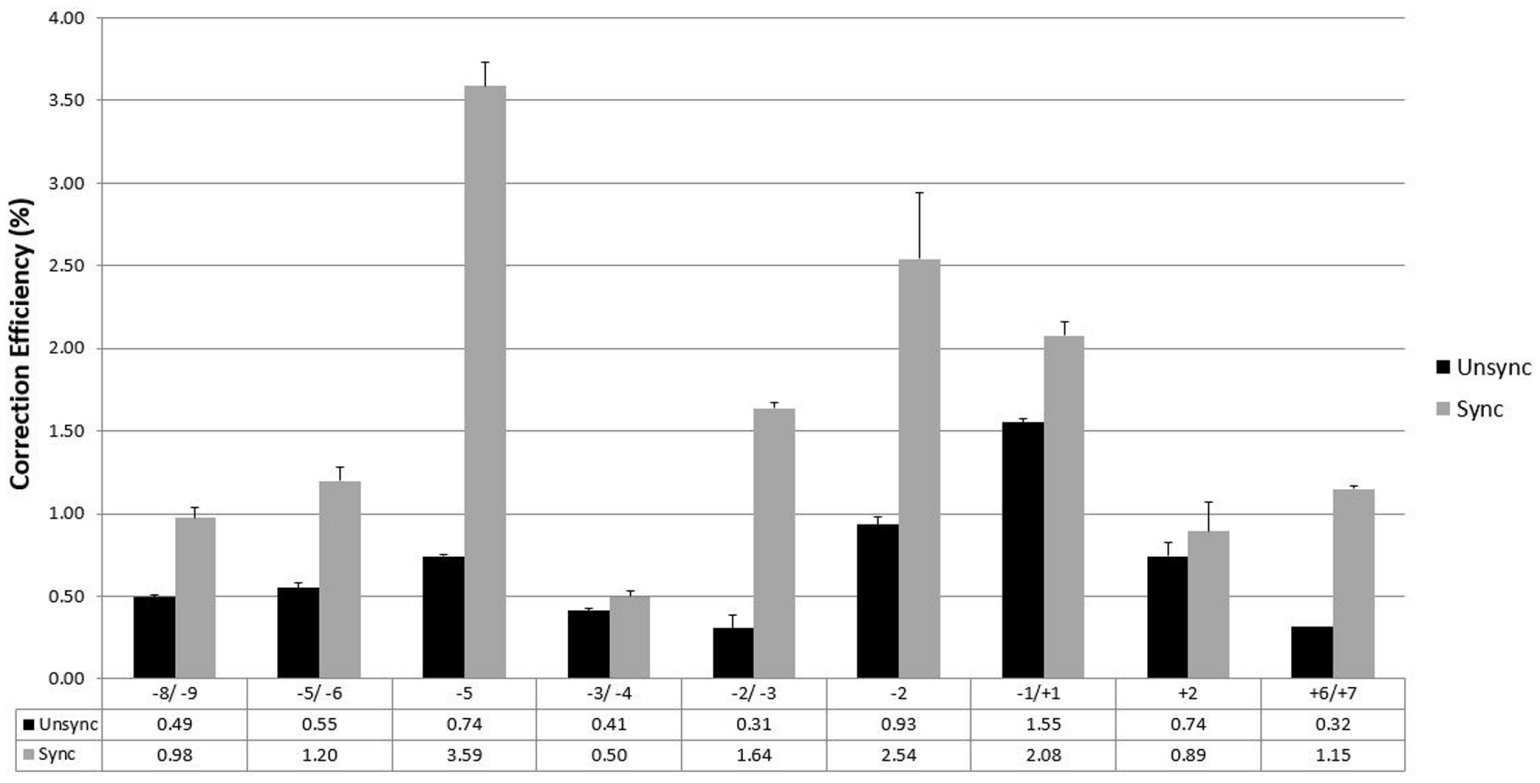
Gene editing of synchronized and released HCT116–19 cells using TALENs and ssODNs. HCT116–19 cells were seeded at 2.5e6 cells in a 100 mm dish and synchronized for 24 hours with 6 uM aphidicolin then released for 4 hours before being electroporated at a concentration of 5e5 cells/100 ul with TALENs and the 72NT ODN under the standard reaction ratio TALEN:ssODN (2 µg/1.35 µg). TALEN amounts reflect the total TALEN plasmid added to each sample in equal portions. Following electroporation, cells were seeded in 6-well plates and allowed to recover for 48 hours and analyses took place on a Guava EasyCyte 5HT flow cytometer (see Materials and Methods). Correction efficiency (%) was determined by the number of viable eGFP positive cells divided by the total viable cells in the population. Each sample set was performed in duplicate and error bars represent standard error.

The frequency of gene editing can be raised if the cells being targeted are progressing through S phase [4] [8]–[10] [29]–[30] [33]–[37]. In a previous study, we demonstrated this phenomenon holds true when ssODNs are paired with TALENs to direct gene repair [21]. We extended that protocol and used the most active pairs of TALENs, as defined in Figure 3, in combination with 72NT. Cells were synchronized for 24 hours then released for 4 hours at which time a pair of TALENs and 72NT were introduced by electroporation. After 48 hours, gene editing activity was assessed and results presented in Figure 4. In every case, targeting synchronized and released cells produces a higher level of gene editing than targeting an unsynchronized population, albeit to various degrees of stimulation. The differences among the cleavage sites range from essentially within experimental error to approximately threefold. Again, we see that the area immediately around the target base −1/+1 produces the highest levels of gene editing. Synchronization and release of cells destined for gene editing can enable a higher degree of activity.

**Figure 5:**
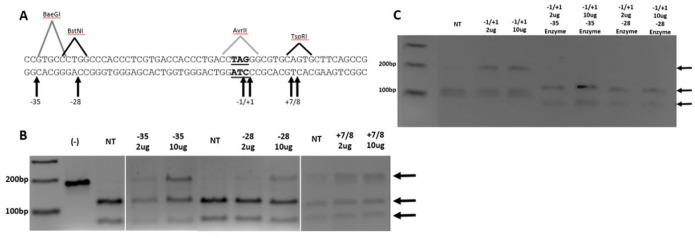
Analyses of inherent TALEN activity within the eGFP gene region. (**A**) Segment of 181 bp PCR product with specific TALEN cut sites indicated by the arrows below and restriction enzyme recognition sequence above. (**B**) 2% TBE agarose gel analysis of RFLP sites created through site-specific TALEN activity. Expected bands are indicated by arrows: 181 bp for active TALENs, 68+113 for BaeGI (−35), 69+112 for BstNI (−28) and 112+69 for TspRI (+7/8). (**C**) 2% TBE RFLP analysis of active gene editing site (−1/+1) and controls. Expected bands are indicated by arrows: 181 bp for active TALENs and 95+86 for AvrII (−1/+1). (−1/+1) treated samples were treated with BaeGI and BstNI enzymes to show specificity of TALEN activity, expected bands are indicated by the lower two arrows (68+113, 70+111).

TALENs are designed to catalyze double-stranded DNA breakage and facilitate gene knockout through NHEJ or, as reported herein, facilitate gene editing. In our hands, the level of TALEN activity required for efficient gene editing is lower than what is traditionally used to enable DNA cleavage for gene knockout. Nevertheless, we sought to evaluate a group of TALENs used in this study for DNA breakage; we chose several TALENs that **do not** support gene editing (−35, −28, +7/+8) and the core TALEN (−1/+1) that supports it the best. Our goal is to ensure that both types of TALENs display activity. One assay system utilizes T7 endonuclease to cleave at heteroduplexed DNA–an outcome of reannealing of DNA strands that arise from TALEN cleavage **and** NHEJ. Our initial pass through all 22 TALEN pairs did not yield robust results except in two important cases (see below). Thus, we used an assay that measures loss of a restriction site (RFLP) as evidence of TALEN activity, as employed convincingly by Bedell et al [27] and Qui et al [38]. Within our targeting zone, we have four sites that correspond to restriction enzyme sites, located at −35, −28, −1/+1 and +7/+8 (see Figure 3) respectively, diagrammed in Figure 5A. Evidence of TALEN activity is the reduction of restriction enzyme cleavage at the designated site. Gene editing was carried out as described in Figure 3 using these four TALENs. The extracted DNA was isolated and amplified across the DNA regions containing sites −35, −28, −1/+1 and +7/+8, then cleaved by BaeGI (−35), BstNI (−28), AvrII (−1/+1) and TspRI (+7/+8) respectively. Figure 5B illustrates the results. In all three cases, TALENs designed for these four sites created a sequence alteration so that a percent of the target DNA is seen to remain resistant to cleavage. The nontreated (NT) lanes display little or no uncut DNA representing the highly efficient activity of the restriction enzyme; a dose dependency (2, 10 µg of TALEN) is also evident. These data demonstrate TALEN activity at sites that do not support gene editing. We also tested the −1/+1 site and the results are displayed in Figure 5C. Again a resistant band appears as a function of TALEN dosage in the reaction. As an extended control to confirm the specificity of this assay, we carried out a restriction enzyme digest with two of the enzymes whose cleavage efficiency and site would not change if TALEN activity was precise at −1/+1. The isolated DNA from treated samples, at −1/+1 was cleaved by BaeGI or BstNI (not AvrII). The results, seen in Figure 5C, show complete cleavage with no residual resistant band. Finally, we were able to obtain robust and reproducible results using the T7 endonuclease assay for sites −1/+1 and +46 (see Figure S1 in File S1). In both cases, TALEN activity was confirmed in a dose-dependent fashion with the predicted band size (459 and 412 bp). Importantly, slightly greater TALEN activity is observed at the +46 site, a site where gene editing activity is undetectable. Hence, by three separate criteria, we find that TALENs supporting or not supporting gene editing exhibit equivalent and significant DNA cleavage activity.

## Discussion

The correction of an integrated, single copy of a mutated eGFP gene has been achieved by the combinatorial activity of ssODNs and TALENs. The repair of the TA**G** stop codon to TA**C** (tyrosine) converts the protein to wild type with phenotypic fluorescence that can be easily quantitated by FACS. While this target gene lacks the clinical relevance of naturally mutated genes, often resulting in inherited disorders, valid outcomes of gene editing can be easily measured at the genetic and protein *activity* level. Previous data from our lab and others [6]–[8] have indicated that double-stranded breaks introduced by the inclusion of anti-cancer drugs in the reaction, enhance the frequency of this repair. While the mechanism of induction still needs more study, a fairly clear picture of its inner workings has emerged and has been confirmed [11] [39].

With the advent of reliable site-specific nucleases operational in mammalian cells, it is now possible to substitute TALENs, Zinc-Finger-Nucleases or CRISPR-Cas9 reagents for the nonspecific activity of anti-cancer drugs. In this study, we have created 22 pairs of TALENs, designed to cut at various places around the TA**G** codon, and paired each with ssODNs designed to direct the repair of the inherent mutant base. Our data suggest that TALENs that cleave at proximal locations near the target base can enhance the frequency of repair to varying degrees. We had previously shown that TALEN activity at −2/−3 (upstream from the mutant base) stimulated the reaction 100 fold above correction levels observed when only the 72NT ssODN was used in this eGFP^−^ targeting system. This choice was somewhat fortuitous since it is one of the most active sites observed when other cleavage site locations were tested. While each TALEN construct contained a workable, established spacer length, some variation in the region is observed and could result from lower levels of cleavage. The TALEN pair built to cleave on either site of the G-target base −1/+1 produced the highest level of correction driven by 72NT. What is also apparent is that proximal cleavage sites are clearly better targets for gene editing as extending the cleavage sites upstream beyond −8/−9 reduces gene editing activity significantly. Thus, somewhere between −8/−9 and −28 respectively activity falls off precipitously. Another interesting feature of this analysis is that the range of productive TALEN target sites appears to be biased leftward, although some activity is observed at the +6/+7 site, downstream. Such a bias may reflect more about the contribution of the 72NT and its mode of action post cleavage, as opposed to direct impact of TALEN activity. Interestingly, substituting the 100-mer (100NT) for 72NT did not rescue the low activity of upstream or downstream sites. A shorter ssODN, 40NT, produced almost undetectable levels of activity throughout the region. Again, it may be that an optimal length of ssODN is required to participate in the gene editing reaction (see below).

A number of other laboratories have clearly demonstrated the productive activity of ssODNs and TALENs to direct genome editing [18] [24]–[28]. In many cases, these experiments were carried out in ES cells, iPSCs or model organisms such as Zebrafish while only a few of them deciphering actual mechanism of action in standard, somatic cell lines have been published. Most of the activity surrounding TALENs center on the generation of knockout cell lines or animals [40]–[42]. Recently, Yang *et al* [18] published an elegant study focusing on cleavage/target site location and several reaction parameters including ssODN length. These workers found that proximal cleavage within 50–100 bases of the target base produced the highest level of gene editing and that there is an optimal length for the ssODN in driving the reaction.

With the advent of tailored nucleases that can cleave at specific sites in the mammalian genome, the pace of development of genome editing toward clinical application has been accelerated. In this work, we employed TALENs in an effort to induce double-stranded DNA breaks, a form of DNA damage that had shown previously to increase the frequency of gene editing directed by ssODNs [19]–[20]. While it is prudent to measure cleavage activity, the most commonly used assay, T7 Endonuclease, has been employed by and large to confirm TALEN activity in studies where the objective was to disable, not repair, a gene. And, in many of these cases, the amount of TALEN needed to execute genomic knockout was 2–5 times higher than the optimal level required for ssODN-directed gene editing. In fact, previous data [21] suggest that increasing amounts of TALENs reduce, not elevate, gene editing activity. We chose to monitor TALEN activity using an assay that identifies the introduction of an RFLP, as reported by Bedell *et al* [27] and Qui *et al* [38]. Within the eGFP gene targeting region, there exists several restriction enzymes cleavage sites that correspond with the cleavage sites of some of our TALENs. We found the assay to be more reliable with less manipulation of the target DNA than the T7 Endonuclease assay. For example, if one surveys the literature, reaction conditions for this assay vary widely suggesting that each modified cell line’s genomic DNA must be treated differently to obtain the desired products [43]–[44]. While we recognize RFLP changes also have a few drawbacks, in our hands this assay produced more reliable, reproducible and robust data. The assay is obviously limited to TALEN cleavage sites that are coincident with restriction enzyme recognition sequences. Our target is a single copy gene in a mammalian genome, thus “gene repair” by HR or “homology-directed repair” (HDR) serves an unlikely mechanism of action. There is always a tendency to assume that biological reactions must occur by the same pathway, a traditional reductionist view, but because of the differences described above we suggest that combinatorial ssODN and TALEN-directed gene editing follows a different route than TALEN-directed genome modification. However, projections of mechanisms of action have to be made with caution. The cell line HCT-116 is genetically devoid of certain MMR functions such as MLH1, MLH3 and PMS2. Therefore correlations among NHEJ, HR, HDR and even MMR in gene editing cannot be definitively established.

The editing frequency of all of the sites tested surrounding the target base are enhanced when synchronized and released cells are used in these experiments. Previously, we had reported this phenomenon with one set of TALENs [21]. These results point again to the importance of DNA replication in the gene editing reaction [1] [4]–[5] [8]. Based on the restrictions we observe regarding the need for proximal cut sites, it is likely that these ds breaks provide an entry point for the ssODN to align in homologous register with the target region. Once aligned, it could provide a 3′OH for extension and act as a “quasi Okazaki fragment” as previously suggested [1]. What complicates this simple explanation is the fact that single-stranded annealing (SSA) and extension synthesis is likely to be in competition with NHEJ. Thus, while gene editing may prove to be successful, by design, the resealed break (by SSA and extension) becomes a newly formed target for TALEN activity and perhaps NHEJ. And, it is unclear how many times this cycle can be repeated. The elegant studies of Liu *et al* [45] however may provide some insight. Building on previous work of the Resnick lab [46], these workers suggest that “SSO-directed information transfer is restricted to the immediate vicinity of the DSB…” This observation predicted the results of the mapping experiments we report in this paper. Liu *et al* [45] also suggest that the SSO may in fact reduce the number of NHEJ events thereby tipping the balance away from the potentially mutagenic activity of NHEJ. In addition, the fact the synchronization and release of these cells enables higher levels of targeting since more cells are traversing through S phase, may shift the balance toward HR or HDR and away from NHEJ. These results also align with the work published by Morozov and Wawrousek [47] in which HR proteins involved in homologous pairing (Rad51 and Rad54) were found to stimulate gene editing while NHEJ proteins KU 70/86 were seen to inhibit the reaction. Shifting the equilibrium toward homology-directed repair (or recombination) may be a fundamental, mechanistic aspect of ssODNs as they direct inheritable changes in the genome. Taking advantage of this biased equilibrium is one area currently under study and is likely to be an important reaction parameter as we define combinatorial methods for utilization in genome modification experiments in mammalian cells.

## Supporting Information

File S1 Figure S1, T7 Endonuclease Assay of TALEN Activity. T7 endonuclease of the eGFP TALEN activity shown at 2 ug and 10 ug TALEN plasmid for the (−1/+1) and (+46) TALEN pairs. Expected bands indicating TALEN activity are indicated by arrows (459 and 412 base pairs respectively).
(TIF)

File S2 DNA Sequence of L848-19 TALEN. DNA sequence of the completed L848-19 TALEN within the pc-GoldyTALEN backbone. RVDs are highlighted in bold.
(DOCX)
