# Supplementary figures and images for: The Position of DNA Cleavage by TALENs and Cell Synchronization Influences the Frequency of Gene Editing Directed by Single-Stranded Oligonucleotides
Source: PLoS One. 2014 May 1;9(5):e96483. doi: 10.1371/journal.pone.0096483 (PMC4006861; doi:10.1371/journal.pone.0096483)

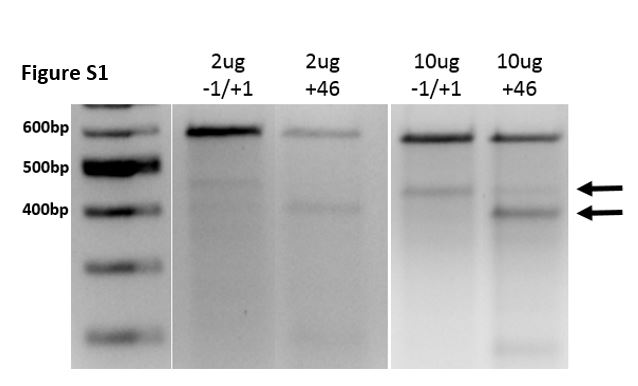

Supplement: File S1 — Figure S1, T7 Endonuclease Assay of TALEN Activity. T7 endonuclease of the eGFP TALEN activity shown at 2 ug and 10 ug TALEN plasmid for the (−1/+1) and (+46) TALEN pairs. Expected bands indicating TALEN activity are indicated by arrows (459 and 412 base pairs respectively). (TIF) [file pone.0096483.s001.tif]
