# Supplementary material for: The Position of DNA Cleavage by TALENs and Cell Synchronization Influences the Frequency of Gene Editing Directed by Single-Stranded Oligonucleotides
Source: PLoS One. 2014 May 1;9(5):e96483. doi: 10.1371/journal.pone.0096483 (PMC4006861; doi:10.1371/journal.pone.0096483)
Supplement: File S2 — DNA Sequence of L848-19 TALEN. DNA sequence of the completed L848-19 TALEN within the pc-GoldyTALEN backbone. RVDs are highlighted in bold. (DOCX) [file pone.0096483.s002.docx]

**Text S2**

tcattaatgcagctgagatcttaagggattactagtttaaacctagttattaatagtaatcaattacggggtcattagttcatagcccatatatggagttccgcgttacataacttacggtaaatggcccgcctggctgaccgcccaacgacccccgcccattgacgtcaataatgacgtatgttcccatagtaacgccaatagggactttccattgacgtcaatgggtggagtatttacggtaaactgcccacttggcagtacatcaagtgtatcatatgccaagtacgccccctattgacgtcaatgacggtaaatggcccgcctggcattatgcccagtacatgaccttatgggactttcctacttggcagtacatctacgtattagtcatcgctattaccatggtcgaggtgagccccacgttctgcttcactctccccatctcccccccctccccacccccaattttgtatttatttattttttaattattttgtgcagcgatgggggcggggggggggggggcgcgcgccaggcggggcggggcggggcgaggggcggggcggggcgaggcggagaggtgcggcggcagccaatcagagcggcgcgctccgaaagtttccttttatggcgaggcggcggcggcggcggccctataaaaagcgaagcgcgcggcgggcgggagtcgctgcgcgctgccttcgccccgtgccccgctccgccgccgcctcgcgccgcccgccccggctctgactgaccgcgttactcccacaggtgagcgggcgggacggcccttctcctccgggctgtaattagcgcttggtttaatgacggcttgtttcttttctgtggctgcgtgaaagccttgaggggctccgggagctagagcctctgctaaccatgttcatgccttcttctttttcctacagctcctgggcaacgtgctggttattgtgctgtctcatcattttggcaaagaattttaagctaggatctaactcgagaaagatattgtatatatcgtaacaataggaggttcaacaatggcttcctcccctccaaagaaaaagagaaaggttagttggaaggacgcaagtggttggtctagagtggatctacgcacgctcggctacagtcagcagcagcaagagaagatcaaaccgaaggtgcgttcgacagtggcgcagcaccacgaggcactggtgggccatgggtttacacacgcgcacatcgttgcgctcagccaacacccggcagcgttagggaccgtcgctgtcacgtatcagcacataatcacggcgttgccagaggcgacacacgaagacatcgttggcgtcggcaaacagtggtccggcgcacgcgccctggaggccttgctcacggatgcgggggagttgagaggtccgccgttacagttggacacaggccaacttgtgaagattgcaaaacgtggcggcgtgaccgcaatggaggcagtgcatgcatcgcgcaatgcactgacgggtgcccccctgaac**ctgaccccggaccaagtggtggctatcgccagcaacaatggcggcaagcaagcgctcgaaacggtgcagcggctgttgccggtgctgtgccaggaccatggcctgaccccggaccaagtggtggctatcgccagcaacaatggcggcaagcaagcgctcgaaacggtgcagcggctgttgccggtgctgtgccaggaccatggcctgactccggaccaagtggtggctatcgccagccacgatggcggcaagcaagcgctcgaaacggtgcagcggctgttgccggtgctgtgccaggaccatggcctgactccggaccaagtggtggctatcgccagccacgatggcggcaagcaagcgctcgaaacggtgcagcggctgttgccggtgctgtgccaggaccatggcctgactccggaccaagtggtggctatcgccagccacgatggcggcaagcaagcgctcgaaacggtgcagcggctgttgccggtgctgtgccaggaccatggcctgaccccggaccaagtggtggctatcgccagcaacattggcggcaagcaagcgctcgaaacggtgcagcggctgttgccggtgctgtgccaggaccatggcctgactccggaccaagtggtggctatcgccagccacgatggcggcaagcaagcgctcgaaacggtgcagcggctgttgccggtgctgtgccaggaccatggcctgactccggaccaagtggtggctatcgccagccacgatggcggcaagcaagcgctcgaaacggtgcagcggctgttgccggtgctgtgccaggaccatggcctgactccggaccaagtggtggctatcgccagccacgatggcggcaagcaagcgctcgaaacggtgcagcggctgttgccggtgctgtgccaggaccatggcctgaccccggaccaagtggtggctatcgccagcaacggtggcggcaagcaagcgctcgaaacggtgcagcggctgttgccggtgctgtgccaggaccatggcctgaccccggaccaagtggtggctatcgccagccacgatggcggcaagcaagcgctcgaaacggtgcagcggctgttgccggtgctgtgccaggaccatggcctgaccccggaccaagtggtggctatcgccagcaacaatggcggcaagcaagcgctcgaaacggtgcagcggctgttgccggtgctgtgccaggaccatggcctgaccccggaccaagtggtggctatcgccagcaacggtggcggcaagcaagcgctcgaaacggtgcagcggctgttgccggtgctgtgccaggaccatggcctgaccccggaccaagtggtggctatcgccagcaacaatggcggcaagcaagcgctcgaaacggtgcagcggctgttgccggtgctgtgccaggaccatggcctgaccccggaccaagtggtggctatcgccagcaacaatggcggcaagcaagcgctcgaaacggtgcagcggctgttgccggtgctgtgccaggaccatggcctgactccggaccaagtggtggctatcgccagccacgatggcggcaagcaagcgctcgaaacggtgcagcggctgttgccggtgctgtgccaggaccatggcctgactccggaccaagtggtggctatcgccagccacgatggcggcaagcaagcgctcgaaacggtgcagcggctgttgccggtgctgtgccaggaccatggcctgaccccggaccaagtggtggctatcgccagcaacattggcggcaagcaagcgctcgaaacggtgcagcggctgttgccggtgctgtgccaggaccatggcctgaccccggaccaagtggtggctatcgccagccacgatggcggcaagcaagcgctcgaaagcattgtggcccagctgagccggcctgatccggcgttggcc**gcgttgaccaacgaccacctcgtcgccttggcctgcctcggcggacgtcctgccatggatgcagtgaaaaagggattgccgcacgcgccggaattgatcagaagagtcaatcgccgtattggcgaacgcacgtcccatcgcgttgcctctagatcccagctagtgaaatctgaattggaagagaagaaatctgaacttagacataaattgaaatatgtgccacatgaatatattgaattgattgaaatcgcaagaaattcaactcaggatagaatccttgaaatgaaggtgatggagttctttatgaaggtttatggttatcgtggtaaacatttgggtggatcaaggaaaccagacggagcaatttatactgtcggatctcctattgattacggtgtgatcgttgatactaaggcatattcaggaggttataatcttccaattggtcaagcagatgaaatgcaaagatatgtcgaagagaatcaaacaagaaacaagcatatcaaccctaatgaatggtggaaagtctatccatcttcagtaacagaatttaagttcttgtttgtgagtggtcatttcaaaggaaactacaaagctcagcttacaagattgaatcatatcactaattgtaatggagctgttcttagtgtagaagagcttttgattggtggagaaatgattaaagctggtacattgacacttgaggaagtgagaaggaaatttaataacggtgagataaacttttaataggagctcgcccggggatctaattcaattagagactaattcaattagagctaattcaattaggatccagaattcattgatgagtttggacaaaccacaactagaatgcagtgaaaaaaatgctttatttgtgaaatttgtgatgctattgctttatttgtaaccattataagctgcaataaacaagttaacaacaacaattgcattcattttatgtttcaggttcagggggaggtgtgggaggttttttaaagcaagtaaaacctctacaaatgtggtcattgatgagtttggacaaaccacaactagaatgcagtgaaaaaaatgctttatttgtgaaatttgtgatgctattgctttatttgtaaccattataagctgcaataaacaagttaacaacaacaattgcattcattttatgtttcaggttcagggggaggtgtgggaggttttttaaagcaagtaaaacctctacaaatgtggtcattcagctggcgtaatagcgaagaggcccgcaccgatcgcccttcccaacagttgcgcagcctgaatggcgaatggaaattgtaagcgttaatattttgttaaaattcgcgttaaatttttgttaaatcagctcattttttaaccaataggccgaaatcggcaaaatcccttataaatcaaaagaatagaccgagatagggttgagtgttgttccagtttggaacaagagtccactattaaagaacgtggactccaacgtcaaagggcgaaaaaccgtctatcagggcgatggcccactacgtgaaccatcaccctaatcaagttttttggggtcgaggtgccgtaaagcactaaatcggaaccctaaagggagcccccgatttagagcttgacggggaaagccggcgaacgtggcgagaaaggaagggaagaaagcgaaaggagcgggcgctagggcgctggcaagtgtagcggtcacgctgcgcgtaaccaccacacccgccgcgcttaatgcgccgctacagggcgcgtcaggtggcacttttcggggaaatgtgcgcggaacccctatttgtttatttttctaaatacattcaaatatgtatccgctcatgagacaataaccctgataaatgcttcaataatattgaaaaaggaagagtatgagtattcaacatttccgtgtcgcccttattcccttttttgcggcattttgccttcctgtttttgctcacccagaaacgctggtgaaagtaaaagatgctgaagatcagttgggtgcacgagtgggttacatcgaactggatctcaacagcggtaagatccttgagagttttcgccccgaagaacgttttccaatgatgagcacttttaaagttctgctatgtggcgcggtattatcccgtattgacgccgggcaagagcaactcggtcgccgcatacactattctcagaatgacttggttgagtactcaccagtcacagaaaagcatcttacggatggcatgacagtaagagaattatgcagtgctgccataaccatgagtgataacactgcggccaacttacttctgacaacgatcggaggaccgaaggagctaaccgcttttttgcacaacatgggggatcatgtaactcgccttgatcgttgggaaccggagctgaatgaagccataccaaacgacgagcgtgacaccacgatgcctgtagcaatggcaacaacgttgcgcaaactattaactggcgaactacttactctagcttcccggcaacaattaatagactggatggaggcggataaagttgcaggaccacttctgcgctcggcccttccggctggctggtttattgctgataaatctggagccggtgagcgtgggtctcgcggtatcattgcagcactggggccagatggtaagccctcccgtatcgtagttatctacacgacggggagtcaggcaactatggatgaacgaaatagacagatcgctgagataggtgcctcactgattaagcattggtaactgtcagaccaagtttactcatatatactttagattgatttaaaacttcatttttaatttaaaaggatctaggtgaagatcctttttgataatctcatgaccaaaatcccttaacgtgagttttcgttccactgagcgtcagaccccgtagaaaagatcaaaggatcttcttgagatcctttttttctgcgcgtaatctgctgcttgcaaacaaaaaaaccaccgctaccagcggtggtttgtttgccggatcaagagctaccaactctttttccgaaggtaactggcttcagcagagcgcagataccaaatactgtccttctagtgtagccgtagttaggccaccacttcaagaactctgtagcaccgcctacatacctcgctctgctaatcctgttaccagtggctgctgccagtggcgataagtcgtgtcttaccgggttggactcaagacgatagttaccggataaggcgcagcggtcgggctgaacggggggttcgtgcacacagcccagcttggagcgaacgacctacaccgaactgagatacctacagcgtgagctatgagaaagcgccacgcttcccgaagggagaaaggcggacaggtatccggtaagcggcagggtcggaacaggagagcgcacgagggagcttccagggggaaacgcctggtatctttatagtcctgtcgggtttcgccacctctgacttgagcgtcgatttttgtgatgctcgtcaggggggcggagcctatggaaaaacgccagcaacgcggcctttttacggttcctggccttttgctggccttttgctcacatgttctttcctgcgttatcccctgattctgtggataaccgtattaccgcctttgagtgagctgataccgctcgccgcagccgaacgaccgagcgcagcgagtcagtgagcgaggaagcggaagagcgcccaatacgcaaaccgcctctccccgcgcgttggccgat
